# Association between serum antinuclear antibody and rheumatoid arthritis

**DOI:** 10.3389/fimmu.2024.1358114

**Published:** 2024-04-22

**Authors:** Fang Liu, Xiu-Qin Wang, Jian-Wen Zou, Ming Li, Cui-Cui Pan, Yuan-Quan Si

**Affiliations:** ^1^ Department of Clinical Laboratory, Shandong Provincial Hospital Affiliated to Shandong First Medical University, Jinan, Shandong, China; ^2^ Department of Rheumatology, Shandong Provincial Hospital Affiliated to Shandong First Medical University, Jinan, Shandong, China

**Keywords:** rheumatoid arthritis, antinuclear antibody, correlation analysis, curve relation, smooth curve

## Abstract

**Background:**

The relationship between serum antinuclear antibody (ANA) and rheumatoid arthritis (RA) remains unknown. Therefore, we aimed to evaluate whether serum ANA was associated with an increased risk of RA in a case–control study.

**Methods:**

Patients with rheumatoid arthritis hospitalized at Shandong Provincial Hospital from January 2018 to December 2022 were recruited as the case group, and patients with other types of arthritis and healthy people at the same time were taken as the control group. Antinuclear antibody (ANA) was detected by indirect immunofluorescence assays. Propensity score matching was employed to construct a cohort of patients exhibiting comparable baseline characteristics. The relationship between serum ANA and the risk of rheumatoid arthritis was analyzed by logistic regression analysis.

**Results:**

A total of 1,175 patients with RA and 1,662 control subjects were included in this study. After adjusting for potential confounding factors in the propensity-score matched cohort, the risk of RA gradually increased with rising of ANA titers. When ANA titers were divided into three groups (1:100, 1:320, and 1:1,000), the OR (95% CI) for ANA titers from low to high was 3.95 (3.01, 5.18), 16.63 (9.44, 29.30), and 17.34 (9.53, 31.54), respectively, compared to those when ANA was negative. The ANA patterns closely related to the occurrence of RA include nuclear homogeneous, nuclear speckled, and cytoplasmic speckled. Among them, the positive rate of nuclear homogeneous was the highest, which accounted for 42.64%. The OR (95% CI) of ANA patterns including nuclear homogeneous, nuclear speckled, and cytoplasmic speckled was 16.81 (11.46, 24.65), 3.40 (2.49, 4.63), and 3.09 (1.77, 5.40), respectively.

**Conclusion:**

There was a curve relation between ANA titer and RA, and the higher the ANA titer, the higher the probability of RA. However, there was no statistical difference in probability of RA for 1:320 versus 1:1,000 ANA titers. The most important kind of ANA pattern in the blood of RA patients was nuclear homogeneous. These findings suggest that ANA may be a novel risk marker for RA.

## Introduction

Rheumatoid arthritis (RA) is recognized as an autoimmune disorder characterized by chronic inflammation that primarily impacts the joints while also being linked to various systemic aberrations of the immune system (1). If inadequately treated, RA can lead to progressive joint deterioration and irreversible impairment (2). Many circulating autoantibodies have been found in the serum of most patients with RA, including rheumatoid factor (RF) and anti-citrullinated protein antibodies (ACPAs) (3, 4).

Antinuclear antibodies (ANAs) comprise a diverse array of autoantibodies that specifically target various nuclear and cytoplasmic components within cells. The detection of ANA is facilitated through the implementation of various immunochemical methods, including indirect immunofluorescence assay (IIFA), enzyme-linked immunosorbent assay (ELISA), multiplex assay, and line immunoassay formats. Notably, the IIFA utilizing HEp-2 cells has long been regarded as the gold standard for ANA detection, providing reliable and accurate results (5, 6).

ANA test results are typically presented in two parts: besides the titer or fluorescence intensity of the antibodies, it also provides the fluorescence pattern produced by these antibodies. The observed fluorescence patterns encompass various cellular components such as the nucleus, cytoplasm, and patterns associated with mitotic cells. In order to establish a standardized nomenclature and definition for ANA, the International Consensus on ANA Patterns (ICAP) initiative has achieved consensus and aims to gradually transition to a more appropriate term: anti-cellular antibodies (ACs). These AC categories consist of 29 distinct staining patterns denoted as AC1-AC29 (7). Each individual staining pattern arises from the presence of one or multiple autoantibodies (8).

As is well known, ANA are important biomarkers for multiple systemic autoimmune diseases, such as systemic lupus erythematosus (SLE), Sjögren’s syndrome (SS), scleroderma (SSc), polymyositis (PM), and mixed connective tissue disease (MCTD) (5, 9). However, the precise clinical implications of ANA in RA and the relationship with other serological markers have remained ambiguous. The current study was conducted with the aim of elucidating the correlation between serum ANA and RA. Meanwhile, several autoantibodies (including CCP and MCV) and acute phase reactants (such as C-reactive protein and erythrocyte sedimentation rate) of the disease were also investigated.

## Materials and methods

### Patients

**Figure 1:**
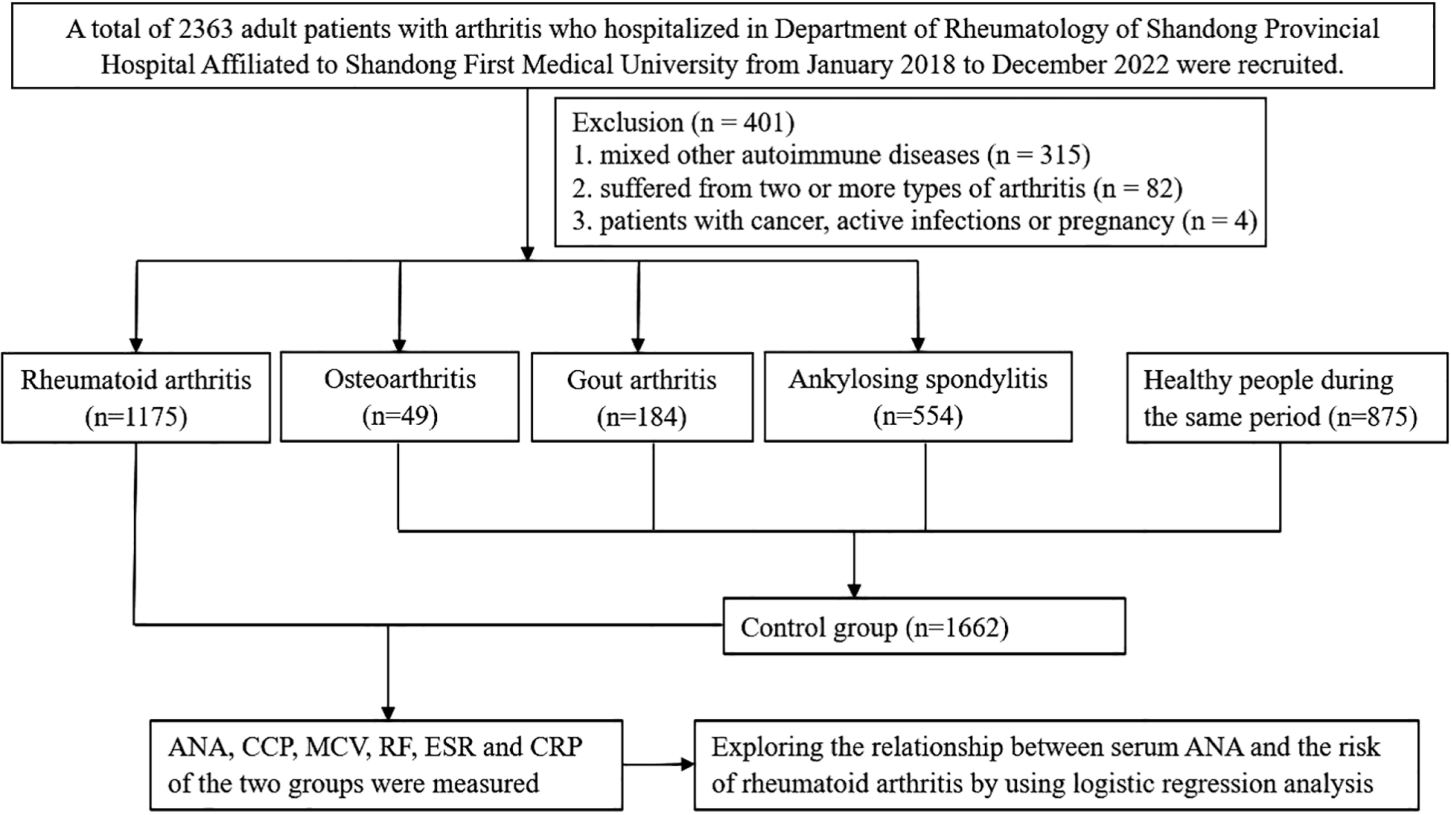
Flowchart showing selection criteria and analysis process of study participants.

We recruited patients with rheumatoid arthritis, which were newly diagnosed according to the 2010 American College of Rheumatology (ACR)/European League Against Rheumatism (EULAR) classification criteria for RA in the Department of Rheumatology of Shandong Provincial Hospital Affiliated to Shandong First Medical University from January 2018 to December 2022 as case group. The control group was composed of patients with other types of arthritis (such as ankylosing spondylitis, gout arthritis, and osteoarthritis) hospitalized in the Department of Rheumatology and healthy people during the same period. Just as shown in **Figure 1**, participants were all over 18 years old. Participants were excluded if they suffered from two or more types of arthritis. Patients with other autoimmune diseases, such as SLE, SS, SSc, PM, and vasculitis, were also excluded from the analyses. All procedures involving human participants were approved by the Shandong Provincial Hospital Affiliated to Shandong First Medical University Research Ethics Committee, and informed consents were obtained from all participants.

### Data collection and blood indicators detection

Demographic variables including age, gender, history of common autoimmune diseases (AID, such as SLE, SS, SSc, PM, and vasculitis) were obtained. Blood examination included antinuclear antibody (ANA), cyclic citrullinated peptide (CCP), rheumatoid factor (RF), erythrocyte sedimentation rate (ESR), C-reactive protein (CRP), and mutant citrulline vimentin (MCV). Elbow venous blood was extracted from all participants after fasting for 6–8 h. ANA levels were determined by an indirect immunofluorescence assay using HEp-2 cells as the substrate by a commercial kit (Euroimmun, Germany). All sections were examined independently by two experienced laboratory staff, and positive and negative control serum samples were included in each run. The analysis was performed for the most prevalent ANA patterns (nuclear homogeneous, nuclear speckled, cytoplasmic speckled, nucleolar, and centromere), and other less common ANA patterns were classified as other patterns. Only monospecific nuclear patterns were included; the primary pattern was selected for two or more patterns. Serum ANA level exceeding 1:100 was seen as positive.

### Statistical analysis

In light of the variances in the baseline characteristics among participants in the two groups (**Table 1**), propensity-score matching (with propensity score in the range of 0.02) was applied to construct a cohort of patients with similar baseline characteristics. Age and sex were matched with the use of a 1:1 matching between RA and non-RA groups. In our research, numerical variables were presented in the form of mean ± standard deviation (SD) or median with interquartile range (IQR). Student’s t-test was employed for assessing normal distributions, while the Mann–Whitney test was utilized for non-normal distributions. Categorical variables, on the other hand, were expressed as frequencies and evaluated using Pearson’s χ^2^ test or Fisher exact test. We evaluated the possible linear and nonlinear relationships between ANA and RA by multivariate linear regression models adjusted for age and gender in the propensity-score matched cohort. Smooth curve fitting was also employed to analyze the independent relationship between them after adjusting the confounding factors. All analyses were performed using Empower Stats software (version 4.1, USA) and R software (http://www.R-project.org). *p* < 0.01 was considered statistically significant.

**Table 1:** Baseline characteristics of the participants before and after propensity-score matching.

Variables	Before Matching (n= 2837)				After Matching* (n= 1196)		
	Non-RA (n = 1662)	RA (n = 1175)	*P* value		Non-RA (n = 598)	RA (n = 598)	*P* value
Age (years)	38.9 (14.4)	57.4 (13.1)	<0.001		51.1 (15.0)	51.4 (13.7)	0.688
Sex			<0.001				0.067
Male	749 (45.07%)	335 (28.51%)			252 (42.14%)	221 (36.96%)	
Female	913 (54.93%)	840 (71.49%)			346 (57.86%)	377 (63.04%)	
CCP (U/mL)	0.5 (0.5-1.8)	198.6 (23.1-200.0)	<0.001		0.5 (0.5-2.1)	192.9 (21.1-215.5)	<0.001
MCV (U/mL)	6.3 (4.4-8.3)	255.5 (25.3-1000.0)	<0.001		6.2 (4.5-8.6)	174.7 (21.3-1000.0)	<0.001
RF (IU/mL)	9.7 (8.9-10.8)	99.2 (15.0-368.5)	<0.001		9.7 (8.9-10.9)	94.8 (13.4-341.5)	<0.001
CRP (mg/L)	15.1 (4.7-38.2)	19.7 (6.2-53.6)	<0.001		15.4 (4.1-41.7)	19.2 (5.3-46.9)	0.06
ESR (mm/hour)	13.0 (6.0-26.0)	50.0 (26.0-78.0)	<0.001		18.0 (8.0-51.0)	45.0 (23.0-73.0)	<0.001
ANA titers			<0.001				<0.001
Negative	1119 (67.33%)	289 (24.60%)			370 (61.87%)	133 (22.24%)	
1:100	502 (30.20%)	534 (45.45%)			198 (33.11%)	281 (46.99%)	
1:320	25 (1.50%)	197 (16.77%)			16 (2.68%)	96 (16.05%)	
1:1000	16 (0.96%)	155 (13.19%)			14 (2.34%)	88 (14.72%)	
ANA patterns			<0.001				<0.001
Negative	1119 (67.33%)	289 (24.60%)			370 (61.87%)	133 (22.24%)	
Nuclear homogeneous	76 (4.57%)	479 (40.77%)			42 (7.02%)	255 (42.64%)	
Nuclear speckled	332 (19.98%)	282 (24.00%)			127 (21.24%)	156 (26.09%)	
Centromere	4 (0.24%)	9 (0.77%)			2 (0.33%)	3 (0.50%)	
Nucleolar	54 (3.25%)	31 (2.64%)			24 (4.01%)	14 (2.34%)	
Cytoplasmic speckled	51 (3.07%)	70 (5.96%)			27 (4.52%)	30 (5.02%)	
Other patterns	26 (1.56%)	15 (1.28%)			6 (1.00%)	7 (1.17%)	

*Age and sex were matched between RA and Non-RA groups.

Abbreviations: RA, rheumatoid arthritis; ANA, antinuclear antibody; CCP, cyclic citrullinated peptide; MCV, mutant citrulline vimentin; RF, rheumatoid factor; CRP, C-reactive protein; ESR, erythrocyte sedimentation rate.

## Results

### Patient selection for subsequent analyses

A total of 2,837 patients met the criteria for this study; 1,175 patients with rheumatoid arthritis were selected as the case group, while 787 patients with other types of arthritis (including 554 ankylosing spondylitis, 184 gout arthritis, and 49 cases of osteoarthritis) and 875 healthy subjects during the same period as the control group. The patient characteristics before and after propensity-score matching are listed in **Table 1**. Before propensity-score matching, there were significant differences between the two groups with regard to age, gender, ANA titers and patterns, CCP, MCV, RF, CRP, and ESR on the basis of available data. There was no difference between RA and non-RA group in terms of age, gender, and CRP after matching, and 38.13% patients were ANA positive in the non-RA group while the positive rate of ANA in patients with RA was 77.76%. Furthermore, the highest percentage of ANA pattern of RA patients was nuclear homogeneous (42.64%).

### Characteristics of ANA-positive patients

To further study the influence of ANA in RA, the characteristics of age, gender, and blood indicators between the ANA-positive and ANA-negative group are described in **Table 2**. CCP, MCV, and RF of patients testing positive for ANA were significantly higher than those of patients testing negative for ANA (all *p* < 0.001). The relationships between ANA and CCP or RF among rheumatoid arthritis patients with or without CCP + or RF + were analyzed by logistic regression analysis. Just as shown in **Supplementary Tables S3**, **S4**, the results suggested that ANA titer was positively correlated with CCP or RF among patients with RA. Similarly, the results also suggested that nuclear homogeneous was significantly associated with CCP or RF among rheumatoid arthritis patients.

**Table 2:** Characteristics of ANA-negative and ANA-positive patients with RA.

Variables	ANA-negative (n = 289)	ANA-positive (n = 886)	*P* value
Age (years)	58.2 (12.7)	57.1 (13.3)	0.245
Sex			0.013
Male	99 (34.26%)	236 (26.64%)	
Female	190 (65.74%)	650 (73.36%)	
CCP (U/mL)	53.8 (2.0-200.0)	200.0 (48.2-200.0)	<0.001
MCV (U/mL)	37.7 (8.4-682.7)	461.6 (50.6-1000.0)	<0.001
RF (IU/mL)	45.7 (10.6-222.5)	130.0 (28.0-380.0)	<0.001
CRP (mg/L)	24.1 (5.7-64.8)	18.9 (6.3-49.1)	0.038
ESR (mm/hour)	48.0 (23.0-77.0)	51.0 (27.0-78.0)	0.351

Abbreviations: RA, rheumatoid arthritis; ANA, antinuclear antibody; CCP, cyclic citrullinated peptide; MCV, mutant citrulline vimentin; RF, rheumatoid factor; CRP, C-reactive protein; ESR, erythrocyte sedimentation rate.

### The nonlinear relation between the ANA and rheumatoid arthritis

**Figure 2:**
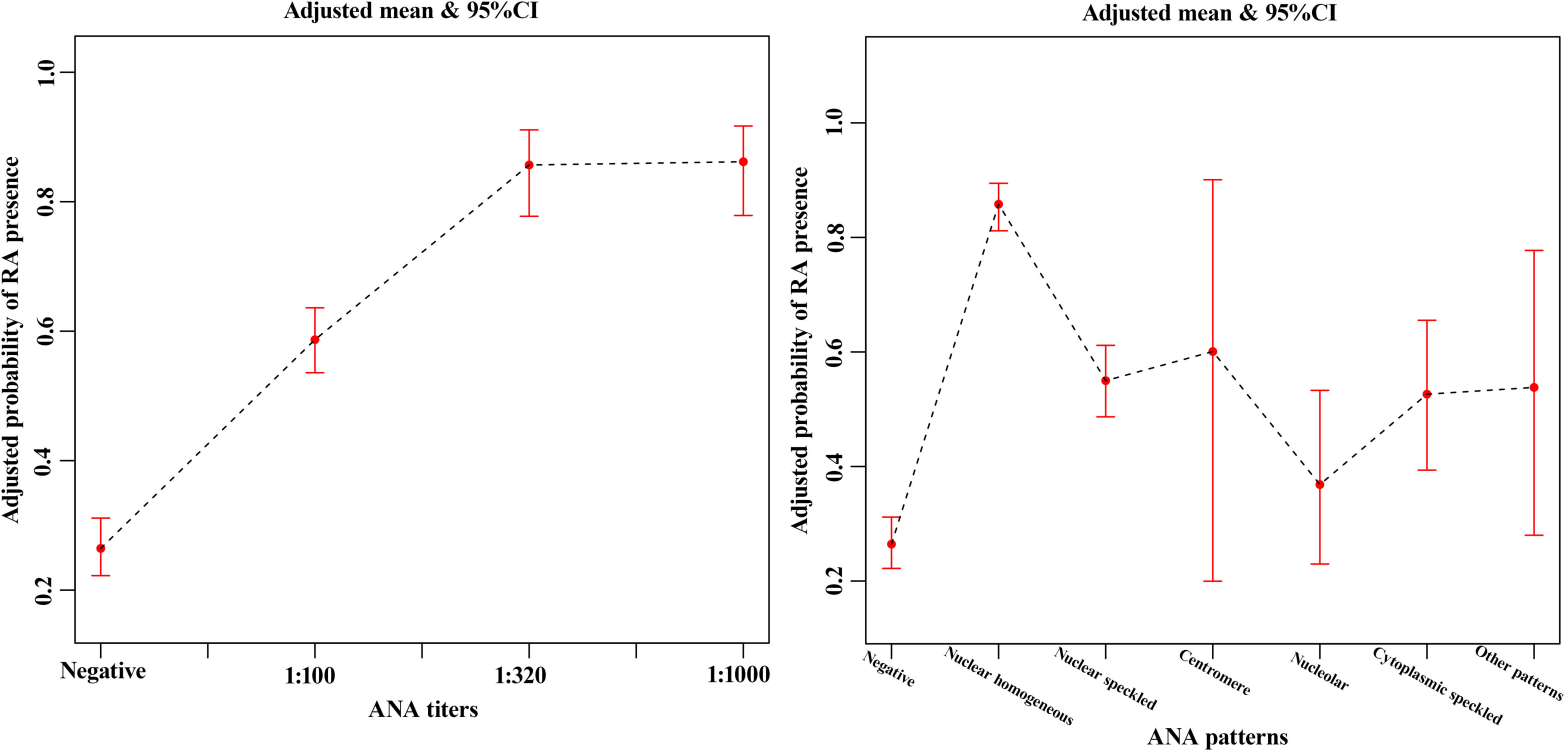
The smooth curve fitting showed the association between ANA titers **(A)**, ANA patterns **(B)** and rheumatoid arthritis after the adjustment of sex and age in the matched cohort. The red lines represented the upper and lower 95% confidence intervals (CIs).

Smooth curve fitting (**Figure 2**) was performed after the adjustment of sex and age in the matched cohort. It was seen from the smooth curves that there existed nonlinear relations between ANA (its titer and pattern) and the probability of rheumatoid arthritis. ANA titer was positively associated with RA, and the higher the ANA titer, the higher the probability of RA. ANA patterns related to the probability of RA included nuclear homogeneous, nuclear speckled, and cytoplasmic speckled, especially nuclear homogeneous.

### Relationship of ANA patterns and titers to rheumatoid arthritis

Logistic regression analysis was performed further in patients with and without RA after matching. In both unadjusted and adjusted (age and sex) logistic regression models, ANA titers were related to rheumatoid arthritis. Compared with those in the ANA-negative patients, the multi-adjusted ORs (95% CIs) of rheumatoid arthritis related to ANA titers (1:100, 1:320, and 1:1,000) were 3.95 (3.01, 5.18), 16.63 (9.44, 29.30), and 17.34 (9.53, 31.54), respectively (**Table 3**). However, there was no statistical difference in probability of RA for 1:320 versus 1:1,000 ANA titers as shown in **Table 4** (*p* = 0.9163). Similarly, the results also suggested that ANA patterns (including nuclear homogeneous, nuclear speckled, and cytoplasmic speckled) were significantly associated with RA in the unadjusted analysis (all *p* < 0.0001). This difference remained statistically significant even after controlling for age and gender. Compared with those in the ANA-negative patients, the multi-adjusted ORs (95% CIs) of rheumatoid arthritis related to ANA patterns mentioned above were 16.81 (11.46, 24.65), 3.40 (2.49, 4.63), and 3.09 (1.77, 5.40), respectively (**Table 3**). We also found that nuclear homogeneous was more significantly associated with rheumatoid arthritis than other ANA patterns except centromere (**Table 4**). According to the reference values of CCP and RF, the participants were categorized as four groups, including CCP−, CCP+, RF−, and RF+ groups. The association between ANA and RA among the four groups with and without matching was analyzed by logistic regression analysis. There was no association between ANA and the risk of RA in the CCP− group, while ANA titer was positively correlated with RA, and the ANA patterns (including nuclear homogeneous and nuclear speckled) were associated with incidence of RA in CCP + group and RF− group, which were basically consistent with above conclusions (**Supplementary Tables S10**, **S11**). The relationships between ANAs and the risk of ankylosing spondylitis, gouty arthritis, or osteoarthritis were analyzed by logistic regression analysis. After adjusting for sex and age, we found that there was no connection between them just as shown in **Supplementary Tables S5**-**S7**.

**Table 3:** Association between ANA positivity and the incidence risk of RA in the propensity-score matched cohort*.

Variables	Non-adjusted		Adjusted I	
	OR (95%CI)	*p*-value	OR (95%CI)	*p*-value
ANA titers				
Negative	Reference		Reference	
1:100	3.95 (3.02, 5.17)	<0.0001	3.95 (3.01, 5.18)	<0.0001
1:320	16.69 (9.49, 29.37)	<0.0001	16.63 (9.44, 29.30)	<0.0001
1:1,000	17.49 (9.62, 31.79)	<0.0001	17.34 (9.53, 31.54)	<0.0001
ANA patterns				
Negative	Reference		Reference	
Nuclear homogeneous	16.89 (11.53, 24.74)	<0.0001	16.81 (11.46, 24.65)	<0.0001
Nuclear speckled	3.42 (2.51, 4.64)	<0.0001	3.40 (2.49, 4.63)	<0.0001
Centromere	4.17 (0.69, 25.25)	0.1198	4.19 (0.69, 25.36)	0.1198
Nucleolar	1.62 (0.82, 3.23)	0.1680	1.62 (0.81, 3.23)	0.1683
Cytoplasmic speckled	3.09 (1.77, 5.39)	<0.0001	3.09 (1.77, 5.40)	<0.0001
Other patterns	3.25 (1.07, 9.83)	0.0373	3.24 (1.07, 9.82)	0.0377

*The propensity-score matched cohort included 598 patients in the RA group and 598 patients in the non-RA group.

RA, rheumatoid arthritis; ANA, antinuclear antibody; OR, odds ratio; 95% CI, 95% confidence interval.

Adjusted I: Adjusted for age, sex.

**Table 4:** Odds ratio (OR) and 95% confidence interval (CI) for the association between ANA and RA in the propensity-score matched cohort*, took 1:320 and nuclear homogeneous as reference, respectively.

Variables	Non-adjusted		Adjusted I	
	OR (95%CI)	*p*-value	OR (95%CI)	*p*-value
ANA titers				
1:320	Reference		Reference	
Negative	0.06 (0.03, 0.11)	<0.0001	0.06 (0.03, 0.11)	<0.0001
1:100	0.24 (0.14, 0.41)	<0.0001	0.24 (0.14, 0.42)	<0.0001
1:1,000	1.05 (0.48, 2.27)	0.9062	1.04 (0.48, 2.26)	0.9163
ANA patterns				
Nuclear homogeneous	Reference		Reference	
Negative	0.06 (0.04, 0.09)	<0.0001	0.06 (0.04, 0.09)	<0.0001
Nuclear speckled	0.20 (0.14, 0.30)	<0.0001	0.20 (0.14, 0.30)	<0.0001
Centromere	0.25 (0.04, 1.52)	0.1319	0.25 (0.04, 1.54)	0.1345
Nucleolar	0.10 (0.05, 0.20)	<0.0001	0.10 (0.05, 0.20)	<0.0001
Cytoplasmic speckled	0.18 (0.10, 0.34)	<0.0001	0.18 (0.10, 0.34)	<0.0001
Other patterns	0.19 (0.06, 0.60)	0.0045	0.19 (0.06, 0.60)	0.0046

*The propensity-score matched cohort included 598 patients in the RA group and 598 patients in the non-RA group.

RA, rheumatoid arthritis; ANA, antinuclear antibody.

Adjusted I: Adjusted for age, sex.

## Discussion

In the present study, our results supported the notion that there was a significant association between ANA and the risk of RA. There were nonlinear relationships between ANA (its titer and pattern) and the incidence of RA. ANA titer was positively correlated with RA. Three ANA patterns (including nuclear homogeneous, nuclear speckled, and cytoplasmic speckled), especially nuclear homogeneous, were associated with increased risk of developing RA.

Less study was focus on the relationship between serum ANA and RA, and the relation between them was still unclear. In those subjects with active RA, ANA positivity was associated with being RF+, especially high titer (10), which was similar to our conclusion. Because we showed that RF of patients testing positive for ANA were significantly higher than that of patients testing negative for ANA. Ishikawa et al. (11) found that ANA was associated with poor treatment response to biological disease-modifying anti-rheumatic drugs (DMARDs) in patients with RA, and they believed ANA as a potential predictor for poor treatment response. Paknikar et al. (12) found that there were significant dissimilarities in patients with rheumatoid arthritis who tested positive and negative for ANA concerning the duration to satisfy the RA criteria and choice of initial pharmacotherapy. Moreover, ANA-positive individuals experienced prolonged duration to fulfill RA criteria. Another study showed that anti-Golgi antibody pattern (one type of ANA patterns) with high titer was closely related to RA (13). These findings could indicate a difference in clinical presentation of patients with RA between ANA positive and ANA negative. Further research was needed to study the association between ANA and RA. To our knowledge, the present study is the first report to clarify the correlation of ANA titer and pattern together with RA.

Thus far, the universally accepted standard for defining the positivity of ANA remains elusive. Previous studies have shown that HEp-2 cell lines derived from cultured human laryngeal epithelial carcinoma exhibit greater sensitivity to the presence of ANA in both patients and controls, when compared to animal tissue sections such as mouse or rat kidney (14). In August 2009, the American College of Rheumatology (ACR) released a recommendation advocating for the utilization of HEp-2 indirect fluorescent antibody in all ANA screenings. Currently, there are two types of screening dilution systems used to determine ANA levels: one utilizes twofold screening dilution systems, including dilutions such as 1:40, 1:80, 1:160, and 1:320. The other employs 3.2-fold screening dilution systems, encompassing dilutions like 1:100, 1:320, 1:1,000, and 1:3,200 (15, 16). Notably, the 1:100 screening dilution has been frequently adopted as the cutoff value in certain clinical assessments (14, 17). In our investigation, patients were subjected to ANA testing using the aforementioned screening dilution systems with HEp-2 cells and monkey liver as substrates, facilitated by a commercial kit. The manufacturers specified reference screening dilutions at four dilutions, namely, 1:100, 1:320, 1:1,000, and 1:3,200. Consequently, the titer exceeding 1:100 was employed as the criterion for defining ANA positivity in our institution. The analyses revealed that the positive rate of ANA in the patients with RA was 45.45%, 16.77%, and 13.19% for a titer of 1:100, 1:320, and 1:1,000, respectively, and no ANA was found with titer higher than 1:1,000. The higher the ANA titer (within the scope from negative to 1:1,000), the higher the probability of RA.

Initially, a consensus encompassing 28 distinct HEp-2 patterns was established, each assigned an alphanumeric code ranging from AC-1 to AC-28 in accordance with the International Consensus on ANA Patterns (ICAP) (18). Subsequent to this initial classification, two additional patterns, AC-29 (19) and AC-0 (negative) (20) were introduced in 2018. Notably, each unique staining pattern observed was attributed to the presence of one or more specific autoantibodies. The most common autoantibodies of nuclear homogeneous pattern included anti-dsDNA antibody (dsDNA), anti-histones antibody (AHA), and anti-nucleosomes antibody (AnuA). Moreover, anti-SS-A antibody (SSA), anti-SS-B antibody (SSB), anti-U1 ribonucleoprotein antibodies (U1RNP), and anti-smith antibody (Sm) were specific autoantibodies of nuclear speckled pattern. Anti-Jo-1 antibody (Jo-1) and anti-ribosomal P protein antibody (Rib-P) were specific autoantibodies of cytoplasmic-speckled pattern (21). In this study, the analysis was performed for the most prevalent ANA patterns including nuclear homogeneous, nuclear speckled, cytoplasmic speckled, nucleolar, and centromere. Other less common ANA patterns were classified as other patterns. We found that ANA patterns related to the risk of RA included nuclear homogeneous, nuclear speckled, and cytoplasmic speckled, especially nuclear homogeneous. The proportion of nuclear homogeneous of RA patients was the highest among all ANA patterns. However, the most common autoantibodies of three kinds of ANA pattern related to RA as described above were negative. This indicated that the antibodies corresponding to ANAs in RA patients were not their common autoantibodies, and the corresponding antibodies were still unclear.

The strengths of our study are the large sample size and well-adjudicated analysis. Our study also has some limitations. First, it tested association, not causation. Furthermore, using a convenience sample from one single institution rather than a population-based study created potential selection bias and limits generalizability. In addition, we lacked data on clinical characteristics of RA, such as disease duration, arthritis distribution, extra-articular symptoms, treatment, and outcomes, which might affect ANA titer (11, 12, 22, 23). Finally, the conclusions are not suitable for CCP-negative patients by our subgroup analysis. More prospective studies are required to assess the importance of this research.

## Conclusions

In conclusion, our results show that high ANA titer may be associated with increased risk of developing RA, so do three ANA patterns (including nuclear homogeneous, nuclear speckled, and cytoplasmic speckled), especially nuclear homogeneous. These findings suggest that ANA may be a novel risk marker for RA; however, future studies investigating the role of ANA in the treatment and outcomes of patients with RA are needed.

## Data availability statement

The raw data supporting the conclusions of this article will be made available by the authors, without undue reservation.

## Ethics statement

The studies involving humans were approved by Ethics Committee of the Shandong Provincial Hospital Affiliated to Shandong First Medical University. The studies were conducted in accordance with the local legislation and institutional requirements. The participants provided their written informed consent to participate in this study.

## Author contributions

YS: Conceptualization, Writing – review & editing. XW: Data curation, Formal analysis, Writing – original draft, Writing – review & editing. FL: Data curation, Formal analysis, Writing – original draft. CP: Software, Writing – original draft. JZ: Data curation, Formal analysis, Writing – original draft. ML: Methodology, Writing – original draft.
