# Supplementary material for: Association between serum antinuclear antibody and rheumatoid arthritis
Source: Front Immunol. 2024 Apr 22;15:1358114. doi: 10.3389/fimmu.2024.1358114 (PMC11070521; doi:10.3389/fimmu.2024.1358114)
Supplement: Supplementary file 1 [file Table_3.docx]

Table S3. Association between ANA positivity and CCP positivity among patients with RA

| Variables | Non-Adjusted | |  | Adjusted I | |
| --- | --- | --- | --- | --- | --- |
|  | OR (95%CI) | *P* value |  | OR (95%CI) | *P* value |
| ANA titers |  |  |  |  |  |
| Negative | Reference |  |  | Reference |  |
| 1:100 | 3.15 (2.15, 4.61) | <0.0001 |  | 3.20 (2.18, 4.70) | <0.0001 |
| 1:320 | 13.87 (5.50, 34.99) | <0.0001 |  | 14.42 (5.70, 36.47) | <0.0001 |
| 1:1000 | 10.95 (4.33, 27.70) | <0.0001 |  | 11.56 (4.55, 29.34) | <0.0001 |
| ANA patterns |  |  |  |  |  |
| Negative | Reference |  |  | Reference |  |
| Nuclear homogeneous | 9.31 (5.43, 15.94) | <0.0001 |  | 9.95 (5.77, 17.15) | <0.0001 |
| Nuclear speckled | 3.16 (1.98, 5.03) | <0.0001 |  | 3.19 (2.00, 5.09) | <0.0001 |
| Centromere | 2.92 (0.36, 23.73) | 0.3162 |  | 2.92 (0.36, 23.87) | 0.3164 |
| Nucleolar | 1.52 (0.60, 3.85) | 0.3763 |  | 1.58 (0.62, 4.01) | 0.339 |
| Cytoplasmic speckled | 2.35 (1.11, 4.97) | 0.0253 |  | 2.22 (1.04, 4.71) | 0.038 |
| Other patterns | 2.37 (0.52, 10.75) | 0.2627 |  | 2.28 (0.50, 10.36) | 0.286 |

The CCP level > 5 U/mL was considered CCP +.

Abbreviations: RA, rheumatoid arthritis; ANA, antinuclear antibody; OR, odds ratio; 95% CI, 95% confidence interval; CCP, cyclic citrullinated peptide.

Adjusted I: Adjusted for age, sex.
