# Supplementary material for: Association between serum antinuclear antibody and rheumatoid arthritis
Source: Front Immunol. 2024 Apr 22;15:1358114. doi: 10.3389/fimmu.2024.1358114 (PMC11070521; doi:10.3389/fimmu.2024.1358114)
Supplement: Supplementary file 2 [file Table_4.docx]

Table S4. Association between ANA positivity and RF positivity among patients with RA

| Variables | Non-Adjusted | |  | Adjusted I | |
| --- | --- | --- | --- | --- | --- |
|  | OR (95%CI) | *P* value |  | OR (95%CI) | *P* value |
| ANA titers |  |  |  |  |  |
| Negative | Reference |  |  | Reference |  |
| 1:100 | 2.04 (1.50, 2.78) | <0.0001 |  | 2.04 (1.50, 2.78) | <0.0001 |
| 1:320 | 6.35 (3.81, 10.59) | <0.0001 |  | 6.40 (3.83, 10.70) | <0.0001 |
| 1:1000 | 3.62 (2.23, 5.88) | <0.0001 |  | 3.69 (2.26, 6.00) | <0.0001 |
| ANA patterns |  |  |  |  |  |
| Negative | Reference |  |  | Reference |  |
| Nuclear homogeneous | 5.25 (3.66, 7.54) | <0.0001 |  | 5.44 (3.77, 7.84) | <0.0001 |
| Nuclear speckled | 1.52 (1.08, 2.15) | 0.0169 |  | 1.52 (1.08, 2.15) | 0.0176 |
| Centromere | 2.70 (0.55, 13.22) | 0.221 |  | 2.70 (0.55, 13.26) | 0.222 |
| Nucleolar | 1.22 (0.57, 2.61) | 0.608 |  | 1.24 (0.58, 2.66) | 0.5839 |
| Cytoplasmic speckled | 1.91 (1.06, 3.42) | 0.0301 |  | 1.82 (1.02, 3.28) | 0.0444 |
| Other patterns | 2.12 (0.66, 6.82) | 0.2078 |  | 2.05 (0.64, 6.62) | 0.2283 |

The RF level > 20 IU/mL was considered RF +.

Abbreviations: RA, rheumatoid arthritis; ANA, antinuclear antibody; OR, odds ratio; 95% CI, 95% confidence interval; RF, rheumatoid factor.

Adjusted I: Adjusted for age, sex.
