# Supplementary material for: Association between serum antinuclear antibody and rheumatoid arthritis
Source: Front Immunol. 2024 Apr 22;15:1358114. doi: 10.3389/fimmu.2024.1358114 (PMC11070521; doi:10.3389/fimmu.2024.1358114)
Supplement: Supplementary file 3 [file Table_5.docx]

Table S5. Association between ANA positivity and the incidence risk of ankylosing spondylitis

| Variables | Non-Adjusted | |  | Adjusted I | |
| --- | --- | --- | --- | --- | --- |
|  | OR (95%CI) | *P* value |  | OR (95%CI) | *P* value |
| ANA titers |  |  |  |  |  |
| Negative | Reference |  |  | Reference |  |
| 1:100 | 0.89 (0.71, 1.13) | 0.3419 |  | 0.98 (0.73, 1.31) | 0.8836 |
| 1:320 | 12.79 (2.92, 55.95) | 0.0007 |  | 17.66 (0.65, 85.49) | 0.1063 |
| 1:1000 | inf. (0.00, Inf) | 0.955 |  | inf. (0.00, Inf) | 0.9646 |
| ANA patterns |  |  |  |  |  |
| Negative | Reference |  |  | Reference |  |
| Nuclear homogeneous | 1.65 (1.00, 2.71) | 0.0487 |  | 2.21 (0.92, 3.99) | 0.0862 |
| Nuclear speckled | 0.98 (0.75, 1.28) | 0.8789 |  | 1.10 (0.78, 1.54) | 0.5846 |
| Centromere | 3.20 (0.29, 35.39) | 0.3432 |  | 4.24 (0.27, 65.68) | 0.3018 |
| Nucleolar | 1.14 (0.63, 2.06) | 0.6581 |  | 0.98 (0.47, 2.05) | 0.9569 |
| Cytoplasmic speckled | 0.69 (0.36, 1.35) | 0.2785 |  | 0.74 (0.33, 1.65) | 0.4576 |
| Other patterns | 0.75 (0.30, 1.85) | 0.5267 |  | 0.81 (0.27, 2.38) | 0.6971 |

Abbreviations: ANA, antinuclear antibody; OR, odds ratio; 95% CI, 95% confidence interval.

Adjusted I: Adjusted for age, sex.
