# Supplementary material for: Association between serum antinuclear antibody and rheumatoid arthritis
Source: Front Immunol. 2024 Apr 22;15:1358114. doi: 10.3389/fimmu.2024.1358114 (PMC11070521; doi:10.3389/fimmu.2024.1358114)
Supplement: Supplementary file 4 [file Table_6.docx]

Table S6. Association between ANA positivity and the incidence risk of gouty arthritis

| Variables | Non-Adjusted | |  | Adjusted I | |
| --- | --- | --- | --- | --- | --- |
|  | OR (95%CI) | *P* value |  | OR (95%CI) | *P* value |
| ANA titers |  |  |  |  |  |
| Negative | Reference |  |  | Reference |  |
| 1:100 | 0.69 (0.48, 1.00) | 0.0486 |  | 0.66 (0.40, 1.11) | 0.1151 |
| 1:320 | 8.87 (1.61, 48.95) | 0.0122 |  | 27.40 (0.26, 2889.53) | 0.1637 |
| 1:1000 | inf. (0.00, Inf) | 0.9749 |  | inf. (0.00, Inf) | 0.9858 |
| ANA patterns |  |  |  |  |  |
| Negative | Reference |  |  | Reference |  |
| Nuclear homogeneous | 0.54 (0.19, 1.54) | 0.2489 |  | 1.39 (0.25, 7.67) | 0.7084 |
| Nuclear speckled | 0.87 (0.58, 1.31) | 0.5118 |  | 0.83 (0.46, 1.48) | 0.5229 |
| Centromere | 4.44 (0.28, 71.38) | 0.2933 |  | 9.78 (0.00, 115.53) | 0.6335 |
| Nucleolar | 0.48 (0.14, 1.59) | 0.2265 |  | 0.33 (0.07, 1.58) | 0.1664 |
| Cytoplasmic speckled | 0.74 (0.28, 1.94) | 0.5398 |  | 0.47 (0.13, 1.68) | 0.2489 |
| Other patterns | 0.89 (0.25, 3.11) | 0.8516 |  | 0.80 (0.15, 4.17) | 0.7934 |

Abbreviations: ANA, antinuclear antibody; OR, odds ratio; 95% CI, 95% confidence interval.

Adjusted I: Adjusted for age, sex.
