# Supplementary material for: Association between serum antinuclear antibody and rheumatoid arthritis
Source: Front Immunol. 2024 Apr 22;15:1358114. doi: 10.3389/fimmu.2024.1358114 (PMC11070521; doi:10.3389/fimmu.2024.1358114)
Supplement: Supplementary file 5 [file Table_7.docx]

Table S7. Association between ANA positivity and the incidence risk of osteoarthritis

| Variables | Non-Adjusted | |  | Adjusted I | |
| --- | --- | --- | --- | --- | --- |
|  | OR (95%CI) | *P* value |  | OR (95%CI) | *P* value |
| ANA titers |  |  |  |  |  |
| Negative | Reference |  |  | Reference |  |
| 1:100 | 1.31 (0.70, 2.45) | 0.3922 |  | 0.61 (0.27, 1.39) | 0.2405 |
| 1:320 | 32.78 (5.26, 204.40) | 0.0002 |  | 24.73 (0.21, 292.00) | 0.1876 |
| 1:1000 | inf. (0.00, Inf) | 0.9774 |  | inf. (0.00, Inf) | 0.9868 |
| ANA patterns |  |  |  |  |  |
| Negative | Reference |  |  | Reference |  |
| Nuclear homogeneous | 3.31 (1.20, 9.15) | 0.021 |  | 1.12 (0.25, 4.95) | 0.8841 |
| Nuclear speckled | 1.23 (0.58, 2.59) | 0.5893 |  | 0.46 (0.17, 1.27) | 0.1358 |
| Centromere | 0.00 (0.00, Inf) | 0.9896 |  | 0.00 (0.00, Inf) | 0.9916 |
| Nucleolar | 2.34 (0.67, 8.18) | 0.1828 |  | 1.22 (0.26, 5.70) | 0.8036 |
| Cytoplasmic speckled | 2.19 (0.63, 7.61) | 0.2196 |  | 1.46 (0.29, 7.27) | 0.6415 |
| Other patterns | 1.46 (0.19, 11.44) | 0.7205 |  | 4.86 (0.50, 47.30) | 0.1732 |

Abbreviations: ANA, antinuclear antibody; OR, odds ratio; 95% CI, 95% confidence interval.

Adjusted I: Adjusted for age, sex.
