# Supplementary material for: Association between serum antinuclear antibody and rheumatoid arthritis
Source: Front Immunol. 2024 Apr 22;15:1358114. doi: 10.3389/fimmu.2024.1358114 (PMC11070521; doi:10.3389/fimmu.2024.1358114)
Supplement: Supplementary file 6 [file Table_8.docx]

Table S8. Association between ANA positivity and the incidence risk of RA among four groups categorized by CCP and RF in the propensity-score matched cohort*

| Variables | CCP -/RF - | |  | CCP -/RF + | |  | CCP +/RF - | |  | CCP +/RF + | |
| --- | --- | --- | --- | --- | --- | --- | --- | --- | --- | --- | --- |
|  | OR (95%CI) | *P* value |  | OR (95%CI) | *P* value |  | OR (95%CI) | *P* value |  | OR (95%CI) | *P* value |
| ANA titers |  |  |  |  |  |  |  |  |  |  |  |
| Negative | Reference |  |  | Reference |  |  | Reference |  |  | Reference |  |
| 1:100 | 1.15 (0.58, 2.25) | 0.6917 |  | 0.49 (0.02, 11.06) | 0.6512 |  | 1.78 (0.97, 3.28) | 0.0646 |  | —§ | — |
| 1:320 | 1.42 (0.16, 12.95) | 0.7542 |  | —§ | — |  | 4.41 (1.27, 15.32) | 0.0197 |  | —§ | — |
| 1:1000 | 1.13 (0.14, 9.45) | 0.907 |  | 2.70 (0.10, 72.10) | 0.5536 |  | 16.09 (1.97, 131.46) | 0.0095 |  | —§ | — |
| ANA patterns |  |  |  |  |  |  |  |  |  |  |  |
| Negative | Reference |  |  | Reference |  |  | Reference |  |  | Reference |  |
| Nuclear homogeneous | 2.13 (0.79, 5.73) | 0.1344 |  | —§ | — |  | 3.34 (1.31, 8.53 | 0.0116 |  | —§ | — |
| Nuclear speckled | 1.01 (0.43, 2.36) | 0.9788 |  | —§ | — |  | 2.37 (1.21, 4.64) | 0.0121 |  | —§ | — |
| Centromere | —§ | — |  | —§ | — |  | —§ | — |  | —§ | — |
| Nucleolar | 0.64 (0.08, 5.15) | 0.6763 |  | —§ | — |  | 1.33 (0.37, 4.79) | 0.6609 |  | —§ | — |
| Cytoplasmic speckled | 0.88 (0.19, 4.09) | 0.8700 |  | —§ | — |  | 3.19 (0.72, 14.15) | 0.1273 |  | —§ | — |
| Other patterns | —§ | — |  | —§ | — |  | 1.13 (0.10, 13.31) | 0.9202 |  | —§ | — |

*The propensity-score matched cohort included 598 patients in the RA group and 598 patients in the Non-RA group.

The CCP level > 5 U/mL or RF level > 20 IU/mL was considered CCP + or RF + respectively.

Abbreviations: RA, rheumatoid arthritis; ANA, antinuclear antibody; OR, odds ratio; 95% CI, 95% confidence interval; CCP, cyclic citrullinated peptide; RF, rheumatoid factor.

Age and sex were adjusted in all analyses.

§: The analysis failed because of the small sample size.
