# Supplementary material for: Association between serum antinuclear antibody and rheumatoid arthritis
Source: Front Immunol. 2024 Apr 22;15:1358114. doi: 10.3389/fimmu.2024.1358114 (PMC11070521; doi:10.3389/fimmu.2024.1358114)
Supplement: Supplementary file 7 [file Table_9.docx]

Table S9. Association between ANA positivity and the incidence risk of RA among four groups categorized by CCP and RF

| Variables | CCP -/RF - | |  | CCP -/RF + | |  | CCP +/RF - | |  | CCP +/RF + | |
| --- | --- | --- | --- | --- | --- | --- | --- | --- | --- | --- | --- |
|  | OR (95%CI) | *P* value |  | OR (95%CI) | *P* value |  | OR (95%CI) | *P* value |  | OR (95%CI) | *P* value |
| ANA titers |  |  |  |  |  |  |  |  |  |  |  |
| Negative | Reference |  |  | Reference |  |  | Reference |  |  | Reference |  |
| 1:100 | 1.16 (0.75, 1.79) | 0.5077 |  | 0.63 (0.10, 3.99) | 0.6261 |  | 1.86 (1.08, 3.20) | 0.0258 |  | —§ | — |
| 1:320 | 0.74 (0.14, 3.87) | 0.7229 |  | —§ | — |  | 5.13 (1.68, 15.67) | 0.0041 |  | —§ | — |
| 1:1000 | 1.36 (0.37, 5.00) | 0.6482 |  | 0.85 (0.04, 19.41) | 0.9205 |  | 25.25 (3.03, 210.70) | 0.0029 |  | —§ | — |
| ANA patterns |  |  |  |  |  |  |  |  |  |  |  |
| Negative | Reference |  |  | Reference |  |  | Reference |  |  | Reference |  |
| Nuclear homogeneous | 1.80 (0.86, 3.77) | 0.1200 |  | —§ | — |  | 3.41 (1.52, 7.64) | 0.0029 |  | —§ | — |
| Nuclear speckled | 0.93 (0.54, 1.58) | 0.7766 |  | —§ | — |  | 2.28 (1.25, 4.17) | 0.007 |  | —§ | — |
| Centromere | 3.35 (0.14, 80.51) | 0.4554 |  | —§ | — |  | 0.48 (0.03, 8.27) | 0.6161 |  | —§ | — |
| Nucleolar | 0.79 (0.26, 2.41) | 0.6789 |  | —§ | — |  | 1.91 (0.56, 6.50) | 0.3007 |  | —§ | — |
| Cytoplasmic speckled | 1.60 (0.65, 3.92) | 0.3034 |  | —§ | — |  | 4.00 (0.98, 16.35) | 0.0538 |  | —§ | — |
| Other patterns | 1.49 (0.30, 7.52) | 0.6260 |  | —§ | — |  | 1.62 (0.23, 11.42) | 0.6268 |  | —§ | — |

The CCP level > 5 U/mL or RF level > 20 IU/mL was considered CCP + or RF + respectively.

Abbreviations: RA, rheumatoid arthritis; ANA, antinuclear antibody; OR, odds ratio; 95% CI, 95% confidence interval; CCP, cyclic citrullinated peptide; RF, rheumatoid factor.

Age and sex were adjusted in all analyses.

§: The analysis failed because of the small sample size.
