# Supplementary material for: Association between serum antinuclear antibody and rheumatoid arthritis
Source: Front Immunol. 2024 Apr 22;15:1358114. doi: 10.3389/fimmu.2024.1358114 (PMC11070521; doi:10.3389/fimmu.2024.1358114)
Supplement: Supplementary file 8 [file Table_10.docx]

Table S10. Association between ANA positivity and the incidence risk of RA among four groups categorized by CCP or RF in the propensity-score matched cohort*

| Variables | CCP - | |  | CCP + | |  | RF - | |  | RF + | |
| --- | --- | --- | --- | --- | --- | --- | --- | --- | --- | --- | --- |
|  | OR (95%CI) | *P* value |  | OR (95%CI) | *P* value |  | OR (95%CI) | *P* value |  | OR (95%CI) | *P* value |
| ANA titers |  |  |  |  |  |  |  |  |  |  |  |
| Negative | Reference |  |  | Reference |  |  | Reference |  |  | Reference |  |
| 1:100 | 1.16 (0.62, 2.19) | 0.6371 |  | 3.76 (2.39, 5.93) | <0.0001 |  | 1.99 (1.34, 2.94) | 0.0006 |  | 4.18 (1.49, 11.75) | 0.0066 |
| 1:320 | 2.42 (0.48, 12.30) | 0.2869 |  | 11.46 (4.40, 29.84) | <0.0001 |  | 4.77 (2.07, 10.97) | 0.0002 |  | —§ | — |
| 1:1000 | 2.02 (0.42, 9.73) | 0.3830 |  | 54.15 (7.34, 399.78) | <0.0001 |  | 5.17 (2.29, 11.70) | <0.0001 |  | 9.58 (1.17, 78.58) | 0.0354 |
| ANA patterns |  |  |  |  |  |  |  |  |  |  |  |
| Negative | Reference |  |  | Reference |  |  | Reference |  |  | Reference |  |
| Nuclear homogeneous | 2.81 (1.16, 6.83) | 0.0227 |  | 15.32 (7.53, 31.17) | <0.0001 |  | 3.77 (2.15, 6.60) | <0.0001 |  | —§ | — |
| Nuclear speckled | 0.98 (0.44, 2.15) | 0.9526 |  | 3.23 (1.91, 5.46) | <0.0001 |  | 2.31 (1.49, 3.56) | 0.0002 |  | —§ | — |
| Centromere | —§ | — |  | 2.01 (0.20, 20.32) | 0.5547 |  | —§ | — |  | —§ | — |
| Nucleolar | 0.60 (0.08, 4.75) | 0.6296 |  | 1.49 (0.53, 4.18) | 0.4443 |  | 1.28 (0.50, 3.26) | 0.6068 |  | —§ | — |
| Cytoplasmic speckled | 1.23 (0.34, 4.41) | 0.7552 |  | 5.74 (1.65, 20.01) | 0.0061 |  | 1.90 (0.84, 4.29) | 0.123 |  | 2.72 (0.32, 22.93) | 0.3571 |
| Other patterns | —§ | — |  | 2.39 (0.47, 12.22) | 0.2961 |  | 1.21 (0.13, 11.00) | 0.8686 |  | 0.87 (0.09, 8.21) | 0.9036 |

*The propensity-score matched cohort included 598 patients in the RA group and 598 patients in the Non-RA group.

The CCP level > 5 U/mL or RF level > 20 IU/mL was considered CCP + or RF + respectively.

Abbreviations: RA, rheumatoid arthritis; ANA, antinuclear antibody; OR, odds ratio; 95% CI, 95% confidence interval; CCP, cyclic citrullinated peptide; RF, rheumatoid factor.

Age and sex were adjusted in all analyses.

§: The analysis failed because of the small sample size.
