# Supplementary material for: Association between serum antinuclear antibody and rheumatoid arthritis
Source: Front Immunol. 2024 Apr 22;15:1358114. doi: 10.3389/fimmu.2024.1358114 (PMC11070521; doi:10.3389/fimmu.2024.1358114)
Supplement: Supplementary file 9 [file Table_11.docx]

Table S11. Association between ANA positivity and the incidence risk of RA among four groups categorized by CCP or RF

| Variables | CCP - | |  | CCP + | |  | RF - | |  | RF + | |
| --- | --- | --- | --- | --- | --- | --- | --- | --- | --- | --- | --- |
|  | OR (95%CI) | *P* value |  | OR (95%CI) | *P* value |  | OR (95%CI) | *P* value |  | OR (95%CI) | *P* value |
| ANA titers |  |  |  |  |  |  |  |  |  |  |  |
| Negative | Reference |  |  | Reference |  |  | Reference |  |  | Reference |  |
| 1:100 | 1.15 (0.77, 1.72) | 0.5040 |  | 3.66 (2.46, 5.43) | <0.0001 |  | 1.74 (1.29, 2.35) | 0.0003 |  | 7.09 (3.13, 16.09) | <0.0001 |
| 1:320 | 1.64 (0.49, 5.56) | 0.4242 |  | 12.43 (5.43, 28.47) | <0.0001 |  | 4.57 (2.16, 9.67) | <0.0001 |  | —§ | — |
| 1:1000 | 1.55 (0.47, 5.07) | 0.4677 |  | 79.04 (10.66, 585.93) | <0.0001 |  | 6.60 (3.12, 13.93) | <0.0001 |  | 23.96 (3.10, 185.26) | 0.0023 |
| ANA patterns |  |  |  |  |  |  |  |  |  |  |  |
| Negative | Reference |  |  | Reference |  |  | Reference |  |  | Reference |  |
| Nuclear homogeneous | 2.22 (1.14, 4.33) | 0.0192 |  | 15.65 (8.55, 28.64) | <0.0001 |  | 3.86 (2.43, 6.13) | <0.0001 |  | —§ | — |
| Nuclear speckled | 0.89 (0.54, 1.46) | 0.6415 |  | 2.93 (1.86, 4.63) | <0.0001 |  | 1.74 (1.24, 2.45) | 0.0015 |  | 3.72 (1.55, 8.94) | 0.0033 |
| Centromere | 3.13 (0.13, 76.49) | 0.4836 |  | 3.22 (0.36, 29.12) | 0.2978 |  | 1.29 (0.19, 8.88) | 0.7961 |  | —§ | — |
| Nucleolar | 1.09 (0.42, 2.81) | 0.8601 |  | 1.93 (0.69, 5.37) | 0.2088 |  | 1.31 (0.64, 2.70) | 0.4605 |  | —§ | — |
| Cytoplasmic speckled | 1.47 (0.65, 3.35) | 0.3539 |  | 7.85 (2.28, 27.05) | 0.0011 |  | 2.29 (1.22, 4.29) | 0.0101 |  | —§ | — |
| Other patterns | 1.24 (0.25, 6.08) | 0.7930 |  | 1.60 (0.47, 5.44) | 0.4528 |  | 1.71 (0.52, 5.66) | 0.3794 |  | 1.00 (0.12, 8.51) | 0.9998 |

The CCP level > 5 U/mL or RF level > 20 IU/mL was considered CCP + or RF + respectively.

Abbreviations: RA, rheumatoid arthritis; ANA, antinuclear antibody; OR, odds ratio; 95% CI, 95% confidence interval; CCP, cyclic citrullinated peptide; RF, rheumatoid factor.

Age and sex were adjusted in all analyses.

§: The analysis failed because of the small sample size.
