# Supplementary material for: Association between serum antinuclear antibody and rheumatoid arthritis
Source: Front Immunol. 2024 Apr 22;15:1358114. doi: 10.3389/fimmu.2024.1358114 (PMC11070521; doi:10.3389/fimmu.2024.1358114)
Supplement: Supplementary file 10 [file Table_12.docx]

Table S12. Baseline characteristics of the participants after propensity-score matching

| Variables | After Matching* (n= 428) | | |
| --- | --- | --- | --- |
|  | Non-RA (n = 214) | RA (n = 214) | *P* value |
| Age (years) | 50.1 (17.4) | 52.4 (13.9) | 0.688 |
| Sex |  |  | 0.544 |
| Male | 73 (34.11%) | 79 (36.92%) |  |
| Female | 141 (65.89%) | 135 (63.08%) |  |
| CCP (U/mL) |  |  | 0.33 |
| ≤ 5 | 126 (58.88%) | 116 (54.21%) |  |
| > 5 | 88 (41.12%) | 98 (45.79%) |  |
| MCV (U/mL) |  |  | 0.75 |
| ≤ 25 | 153 (71.50%) | 150 (70.09%) |  |
| > 25 | 61 (28.50%) | 64 (29.91%) |  |
| RF (IU/mL) |  |  | 0.279 |
| ≤ 15 | 187 (87.38%) | 194 (90.65%) |  |
| > 15 | 27 (12.62%) | 20 (9.35%) |  |
| CRP (mg/L) |  |  | 0.339 |
| ≤ 5 | 67 (31.31%) | 58 (27.10%) |  |
| > 5 | 147 (68.69%) | 156 (72.90%) |  |
| ESR (mm/hour) |  |  | 0.518 |
| ≤ 15 | 63 (29.44%) | 57 (26.64%) |  |
| > 15 | 151 (70.56%) | 157 (73.36%) |  |
| ANA titers |  |  | <0.001 |
| Negative | 130 (60.75%) | 90 (42.06%) |  |
| 1:100 | 71 (33.18%) | 86 (40.19%) |  |
| 1:320 | 8 (3.74%) | 25 (11.68%) |  |
| 1:1000 | 5 (2.34%) | 13 (6.07%) |  |
| ANA patterns |  |  | 0.004 |
| Negative | 130 (60.75%) | 90 (42.06%) |  |
| Nuclear homogeneous | 20 (9.35%) | 43 (20.09%) |  |
| Nuclear speckled | 47 (21.96%) | 55 (25.70%) |  |
| Centromere | 1 (0.47%) | 1 (0.47%) |  |
| Nucleolar | 8 (3.74%) | 9 (4.21%) |  |
| Cytoplasmic speckled | 6 (2.80%) | 13 (6.07%) |  |
| Other patterns | 2 (0.93%) | 3 (1.40%) |  |

*Age, sex, CCP, MCV, RF, CRP and ESR were matched between RA and Non-RA groups. The propensity-score matched cohort included 214 patients in the RA group and 214 patients in the Non-RA group.

Abbreviations: RA, rheumatoid arthritis; ANA, antinuclear antibody; CCP, cyclic citrullinated peptide; MCV, mutant citrulline vimentin; RF, rheumatoid factor; CRP, C-reactive protein; ESR, erythrocyte sedimentation rate.
