# Supplementary material for: Association between serum antinuclear antibody and rheumatoid arthritis
Source: Front Immunol. 2024 Apr 22;15:1358114. doi: 10.3389/fimmu.2024.1358114 (PMC11070521; doi:10.3389/fimmu.2024.1358114)
Supplement: Supplementary file 11 [file Table_13.docx]

Table S13. Association between ANA positivity and the incidence risk of RA in the propensity-score matched cohort*

| Variables | Non-Adjusted | |  | Adjusted I | |
| --- | --- | --- | --- | --- | --- |
|  | OR (95%CI) | *P* value |  | OR (95%CI) | *P* value |
| ANA titers |  |  |  |  |  |
| Negative | Reference |  |  | Reference |  |
| 1:100 | 1.75 (1.16, 2.65) | 0.008 |  | 1.68 (1.10, 2.57) | 0.0159 |
| 1:320 | 4.51 (1.95, 10.46) | 0.0004 |  | 4.48 (1.93, 10.39) | 0.0005 |
| 1:1000 | 3.76 (1.29, 10.90) | 0.015 |  | 3.91 (1.34, 11.44) | 0.0128 |
| ANA patterns |  |  |  |  |  |
| Negative | Reference |  |  | Reference |  |
| Nuclear homogeneous | 3.11 (1.71, 5.63) | 0.0002 |  | 3.15 (1.73, 5.73) | 0.0002 |
| Nuclear speckled | 1.69 (1.05, 2.71) | 0.0297 |  | 1.64 (1.01, 2.66) | 0.0439 |
| Centromere | 1.44 (0.09, 23.40) | 0.7958 |  | 1.37 (0.08, 22.48) | 0.827 |
| Nucleolar | 1.63 (0.60, 4.37) | 0.3362 |  | 1.59 (0.59, 4.30) | 0.3613 |
| Cytoplasmic speckled | 3.13 (1.15, 8.54) | 0.0259 |  | 2.90 (1.05, 7.97) | 0.0394 |
| Other patterns | 2.17 (0.35, 13.23) | 0.4023 |  | 1.93 (0.31, 11.89) | 0.4799 |

*Age, sex, CCP, MCV, RF, CRP and ESR were matched between RA and Non-RA groups. The propensity-score matched cohort included 214 patients in the RA group and 214 patients in the Non-RA group.

Abbreviations: RA, rheumatoid arthritis; ANA, antinuclear antibody; OR, odds ratio; 95% CI, 95% confidence interval; CCP, cyclic citrullinated peptide; MCV, mutant citrulline vimentin; RF, rheumatoid factor; CRP, C-reactive protein; ESR, erythrocyte sedimentation rate.

Adjusted I: Adjusted for age, sex.
